# Conceptual framework to provide culturally congruent care to epilepsy patients in selected rural communities in South Africa

**DOI:** 10.4102/hsag.v29i0.2763

**Published:** 2024-12-11

**Authors:** Muofheni Nemathaga, Maria S. Maputle, Lufuno Makhado, Ntsieni S. Mashau

**Affiliations:** 1 Department of Advanced Nursing Science, Faculty of Health Sciences, University of Venda, Thohoyandou, South Africa; 2 Department of Public Health, Faculty of Health Sciences, University of Venda, Thohoyandou, South Africa

**Keywords:** conceptual framework, culturally congruent, epilepsy care, people living with epilepsy

## Abstract

**Background:**

Epilepsy is a neurological disorder affecting millions of people in Africa. Among other reported findings, many people living with epilepsy (PLWE) believe that the condition is caused by spiritual factors. Previous studies have revealed that majority of PLWE are not receiving adequate care and treatment because of diverse cultural beliefs associated with epilepsy. Consequently, they consult either faith-based healers or traditional healers. Others, however, acknowledge the medical causes of epilepsy and seek developed medical treatment.

**Aim:**

To develop a culturally congruent care conceptual framework to provide comprehensive and holistic epilepsy care.

**Setting:**

This study was conducted in the selected rural communities of Limpopo and Mpumalanga provinces.

**Methods:**

A qualitative multi-method research approach was employed and sub-divided into three phases. Phase 1: All 15 articles were reviewed and appraised using the Critical Appraisal Skills Programme (CASP) rating tool. Phase 2 involved two stages of empirical study in which an exploratory-descriptive study design was used. The study population comprised of 20 traditional healers, 15 faith-based healers, 20 professional nurses and 22 PLWE. Data were collected using in-depth individual interviews and analysed through eight Tesch’s steps of data analysis. Following data analysis, Phase 3 entailed synthesising the systematic and empirical findings into a conceptual framework.

**Results:**

Three themes that emerged from the findings were cultural epilepsy interventions, faith-based epilepsy intervention and medically developed epilepsy interventions.

**Conclusion:**

Incorporation of cultural beliefs, values and practices into the medically developed management of epilepsy is crucial for providing culturally congruent care that is preferred by majority of PLWE.

**Contribution:**

Healthcare providers may tailor care interventions that are culturally appropriate and acceptable hence promoting early diagnosis and treatment adherence leading to improved quality of life.

## Introduction

Epilepsy is a neurological disorder that affects approximately 50 million people globally (Usman, Khalid & Aslam 2020). It is characterised by recurrent seizures, which are brief periods of involuntary movement that may include certain parts of the body (partial) or the full body (generalised), and is occasionally followed by loss of consciousness and control over bowel or bladder function (World Health Organization [WHO] 2022). Epilepsy can be life-threatening if not managed appropriately (WHO 2022).

The management of epilepsy requires a holistic approach that considers cultural beliefs and practices (Ademilokun & Agunbiade 2022; Chabangu et al. 2022). Transcultural nursing acknowledges the significance of cultural beliefs and practices in healthcare provision, thus playing a vital role in bridging the gap between traditional healers, faith-based healers and health professionals in the management of epilepsy.

Traditional healers have been an integral part of society for centuries. They possess in-depth knowledge regarding medicinal plants and traditional remedies that can be used to manage epilepsy (Birhan 2022). Furthermore, their holistic approach to healthcare considers not only the physical but also the spiritual aspects of a patient’s well-being. Faith-based healers depend on spiritual beliefs and practices to provide care for people living with epilepsy (PLWE). They offer prayers, perform rituals and provide emotional support to PLWE and their caregivers. Furthermore, these healing approaches can complement Western medicine prescribed by health professionals (Auditeau et al. 2018; Mesraoua et al. 2021).

Unlike traditional healers, health professionals bring scientific knowledge and evidence-based practices into the management of epilepsy. They diagnose the condition, prescribe appropriate medications, monitor treatment progress and educate patients about self-management strategies (Chowdhury et al. 2020; Seda 2019). According to Leininger (1991), transcultural nursing acknowledges that each cultural group has unique beliefs about illness causation, treatment options and healing practices. Therefore, by integrating traditional healers’ knowledge with faith-based healing approaches and modern medical interventions, transcultural nursing promotes a comprehensive approach and culturally congruent solution to the management of epilepsy.

One key difference between these approaches is the level of scientific evidence supporting them. Medical interventions are backed by extensive research and clinical trials, while traditional and faith-based healing methods often lack this level of empirical support (Kaddumukasa et al. 2021; Shlobin et al. 2022; Zhu et al. 2023). While both traditional and faith healers have been known to provide care for epileptic patients, they often lack the scientific evidence, knowledge and training required to manage the condition effectively. As noted by Keikelame and Swartz (2015), traditional healers have been acknowledged as essential epilepsy care providers because of their ability to provide guidance and their role as custodians of culture. Health professionals, on the other hand, possess the necessary medical expertise but may not always be able to understand or incorporate cultural beliefs and practices into their treatment plans.

Differences in beliefs and practices potentially give rise to conflicts in management and caring between these groups. Health professionals may not recognise the efficacy of traditional or faith healing methods, while traditional or faith healers may not trust modern medicine. Establishing a mutual understanding of epilepsy management across the three groups through educational initiatives that highlight diverse perspectives is crucial. Similarly, a previous study conducted by Nemathaga et al. (2023) revealed that epilepsy management can be greatly improved through collaboration; hence, improving the quality of life of PLWE. Therefore, this article aims to develop a conceptual framework to explain the relationship among providers of epilepsy care to provide culturally congruent interventions for PLWE.

### Theoretical context

This article adopted Leininger’s transcultural nursing theory because it focusses on the diversity of cultures, caring behaviours, health and illness values, beliefs and patterns of behaviour (Leininger & McFarland 2002). This theory guided the study of different cultures and caring behaviours, which would then assist in the development of culturally congruent interventions for PLWE. The major concepts and definitions of transcultural nursing theory are as follows:

### Transcultural nursing

Transcultural nursing is defined as an area of nursing that focusses on relative studies and the analysis of cultures (Leininger & McFarland 2002). Transcultural nursing recognises the importance of cultural diversity in healthcare. In this study, transcultural nursing emphasises the need for healthcare professionals to understand and respect the beliefs, values and practices of different cultures to provide effective care. The key concepts emanating from transcultural nursing were diverse cultural beliefs, values and practices.

### Ethno-nursing

This is the study of nursing care beliefs, values and practices as viewed and understood by a designated culture through its lived experience, beliefs and value system (Leininger & McFarland 2002). In this context, ethno-nursing refers to incorporating cultural beliefs, values and practices into healthcare delivery to ensure the provision of culturally sensitive care that respects patients’ diversity. The key concepts identified from ethno-nursing were cultural beliefs, values, practices and diversity.

### Culturally congruent (nursing) care

Culturally congruent (nursing) care entails cognitively grounded, helpful, supportive, facilitative, or enabling acts or choices and lifeways that offer or support meaningful, valuable and fulfilling health care or welfare services (Leininger & McFarland 2002). ‘Lifeways’ refers to the patterns of living, way of life, customs and practices that characterise a particular culture or ethnic group. In this study, cultural congruence refers to the alignment of patient’s cultural beliefs, values and practices with those of the healthcare provider, thereby ensuring provision of care that is respectful and effective. Comprehensive holistic care, culturally congruent care and cultural sensitivity were the key concepts emanating from culturally congruent (nursing) care.

### Health

Health is a state of well-being that is culturally defined, valued and practised, which considers the capability of people to carry out their everyday activities in culturally articulated, valuable and patterned lifeways (Leininger & McFarland 2002). In this context, health is viewed as holistic and encompasses the physical, mental, emotional and spiritual well-being of PLWE. The key concepts identified were state of well-being and holistic health care.

### Human beings

According to Leininger and McFarland (2002), while human beings are concerned for their own needs, they are inherently compassionate and considerate of others’ wellbeing, needs and continued existence. Leininger and McFarland (2002) described human beings as complex creatures with unique cultural backgrounds, beliefs, values and practices. The key concepts of interest from the perspective of human beings were cultural beliefs, values and practices. Consideration of cultural beliefs, values and practices leads to cultural awareness and the provision of comprehensive and holistic culturally congruent care.

## Research methods and design

A qualitative multi-method research approach was employed and sub-divided into three phases. *Phase 1* was a systematic review that was conducted to determine the effectiveness of available epilepsy interventions in a global context. The search strategy employed was performed in collaboration with the University of Venda’s Faculty of Health Sciences librarian. Articles were searched using ScienceDirect, EBSCOhost, PubMed and Google Scholar. The keywords used were cultural, congruent, epilepsy, interventions and programmes. A total of 15 articles were reviewed and appraised using the Critical Appraisal Skills Programme (CASP) rating tool.

*Phase 2* comprised of two stages in which an exploratory-descriptive study design was used. The study population was comprised of 20 traditional healers, 15 faith-based healers, 20 professional nurses and 22 PLWE. Data were collected using in-depth individual interviews and analysed through eight Tesch’s steps of data analysis (De Vos et al. 2011).

Following data analysis, *Phase 3* entailed synthesising the systematic and empirical findings into a conceptual framework. Three themes that emerged from the findings were cultural epilepsy interventions, faith-based epilepsy interventions and medically developed epilepsy interventions, wherein the major concepts and definitions of transcultural nursing theory were integrated.

### Ethical considerations

The study approval was granted by The Human and Clinical Trails Research Ethics Committee (HCTREC) SHS/19/PH/37/2101.

## Results

The results of 15 articles reviewed and appraised, and the narrative data from the empirical study were used to synthesise the relationship between different care provided for epilepsy care.

### Theme 1: Cultural interventions

Traditional healers were consulted regularly throughout, with at least one traditional healer in every rural community, compared with only one medical doctor or neurologist in the health facility. Utilisation of traditional healers is common throughout the sub-Saharan countries. This was supported by the following quote:

‘PLWE should consult us first; then we will diagnose and treat the disease if it requires spiritual treatment, then we will refer the patients to the hospital for further management. The doctors should also refer the clients if they cannot treat the disease medically.’ (Participant 3, male, 55 years old)

Traditional healers were viewed to offer primary care for people experiencing convulsions and were considered the leading source of information. Some community members in South Africa, regarded traditional healers as pillars of epilepsy management because they can offer counselling.

Many PLWE believed that epilepsy was caused by *gonono* [a fly], witchcraft, a snake in the stomach, urine contents, heredity, evil spirits, demonic possession, difficulty breathing, a curse or punishment, or foam in the lungs, and that epilepsy is contagious. However, other PLWE believed that epilepsy is caused by a disturbance in the brain and genetic disorders. These beliefs influence how epilepsy is perceived; hence, affecting treatment preference. Participant 4 from Limpopo reported:

‘I mean that all the waste in the body forms “mutambuluwo” [*urine*] causes epilepsy. That is why we use urine to cure epilepsy. The illness is caused by the “mutambuluwo” [*urine*]. The illness starts from the body and then travels to the brain.’ (Participant 4, Female, 56 years old)

Traditional healing is usually preferred over developed treatment, contributing significantly to delays in diagnosis and treatment of epilepsy, resulting in poor management of the condition and consequently reducing the patient’s quality of life.

### Theme 2: Faith-based interventions

The findings revealed that faith-based interventions were preferred over medically developed interventions by PLWE in managing their condition. This preference was based on the beliefs that epilepsy is caused by spiritual factors, such as demonic possession, witchcraft, punishment from God for sins and evil spirits.

Faith-based interventions include prayer, meditation, fasting, anointing oil, holy water and prophetic deliverance. People living with epilepsy frequently seek interventions first from faith-based healers, which is unsurprising given that community believe seizures may be caused by spiritual influences, such as ancestral determinism, evil spirits, witchcraft or demonic possession. Participant from Mpumalanga verbalised:

‘There are many things that can cause epilepsy, firstly you don’t sit on “ecansini” (Reed mat) of a person with epilepsy because it’s contagious, and you don’t eat food that was left by the epileptic patient.’ (Participant 1, male, 60 years old)

### Theme 3: Western interventions of epilepsy

While the majority of PLWE combine traditional remedies and medically developed treatment, traditional remedies are often given precedence. The lack of integration between medically developed and traditional healing contributes to the unsatisfactory management of epilepsy. Although some PLWE do consider developed medical management, adherence to the prescribed treatment regimen is often a challenge, which can lead to suboptimal management of the condition.

Health professionals experience challenges at times when managing epilepsy because some PLWE consult faith-based healers and traditional healers as first preference. They only consult local clinics at a later stage. However, health professionals are willing to explore the diverse cultural beliefs to tailor their interventions to accommodate all PLWE. The following quote depicts the findings:

‘Sometimes the management of seizures is difficult because patients consult at a later stage. Some of them disclose that they consult traditional healers and use traditional remedies as preferred and supported by their families. When the seizures are not controlled or become persistent, that is when they consult at the clinic, and it only delays them from receiving appropriate treatment.’ (Participant 7, female, 35 years old)

People living with epilepsy believe that the management of epilepsy can be effective if their cultural beliefs, values and practices are aligned with their treatment plan, thereby promoting adherence. One participant indicated that they had their own management practices to control seizures, but they are open to collaborating with Western medicine as elaborated in the following quote:

‘There is nothing wrong with Western medicine. I advise people living with epilepsy to drink light tea and fresh milk and take medicine from the clinic. The light tea assists to induce vomiting which aims to excrete the foam which causes epilepsy.’ (Participant 12, female, 46 years old)

A professional nurse shared that they do provide *transcultural nursing* when saying:

‘People living with epilepsy have different beliefs on the causes of epilepsy. I am quite aware that some of them consult traditional healers for treatment, so if we work in collaboration, we can encourage them to consult at the clinic to promote treatment adherence.’ (Participant 9, male, 35 years old)

He further said:

‘It is important to learn about PLWE’s cultural beliefs and practices so that we can be able to provide care that is suitable for them.’

Medically, epilepsy is treated with anti-epileptic drugs (AED) with Phenobarbital being the most widely used AED in most countries.

## Discussion

### Interventions for epilepsy care

The study findings showed relationship and limited collaboration in interventions to providing epilepsy care. Cultural beliefs have a significant impact on shaping cultural values and practices regarding epilepsy management (Chabangu et al. 2022; Gosain & Samanta 2022). These beliefs influence not only how PLWE as human beings are managed but also determine the choice of interventions used for their care. For instance, those who believe in spiritual causes seek treatment from traditional or faith-based healers. Those who believe in medical causes consult developed medicine as their first line of treatment. Moreover, cultural beliefs and values influence the practices of epilepsy management by traditional and faith-based healers. According to numerous studies conducted in sub-Saharan Africa, the majority of the population (85%) seek assistance from traditional healers to diagnose and treat their diseases (Adewumi, Oladipo & Adewuya 2020; Anand et al. 2019; Wagner et al. 2020).

Some cultures may have diverse views on the causes of epilepsy or management approaches. Therefore, by acknowledging these differences and incorporating them into the treatment plan, PLWE may feel more comfortable and confident in managing the condition. Moreover, incorporating cultural beliefs can also assist in improving their quality of life. Studies indicate that PLWE who receive culturally congruent care are more likely to adhere to treatment plans and experience improved overall health outcomes (Joo & Liu 2021; Kaihlanen, Hietapakka & Heponiemi 2019; Nemathaga et al. 2023). Similarly, Siriba (2014) revealed that cultural beliefs significantly impact the healthcare-seeking behaviours of PLWE and their caregivers. These cultural factors frequently lead PLWE to consult traditional healers instead of Western-trained healthcare professionals (Siriba 2014). Therefore, it is crucial for healthcare professionals to be educated on diverse cultural beliefs and values, enabling them to provide culturally congruent and holistic care for PLWE. At the same time, traditional and faith-based healers need to receive training in developed medical treatment management to establish effective collaboration aimed at providing comprehensive and holistic interventions for PLWE.

Through fostering collaboration between these various approaches, the spirit of ubuntu – which embodies interconnectedness, compassion and mutual respect within communities – can bridge the gap between traditional practices and developed medicine. Healthcare providers can benefit from the knowledge of herbal medicines and spiritual practices held by traditional healers, while also educating them in the scientific basis of the causes, symptoms and available treatments for epilepsy (Ademilokun and Agubiade 2022). Such collaboration encourages cultural sensitivity by respecting indigenous practices while ensuring PLWE receives evidence-based interventions.

Traditional healers, faith-based healers and health professionals are willing to explore epilepsy management from various perspectives and collaborate to ensure culturally congruent care for the PLWE. Comprehensive training in epilepsy management from diverse perspectives and encouraging collaboration are essential for acquiring new knowledge and skills regarding the causes and management of epilepsy. This approach promotes a better understanding of the illness, ultimately leading to an improvement in quality of life, which is the primary objective. As noted by Solera-Deucher (2021), both traditional healers and Western-trained healthcare professionals acknowledge the benefits of integration, which can lead to the provision of more culturally sensitive care for PLWE, thus improving the management of epilepsy. Moreover, the WHO highlights the significance of incorporating cultural beliefs, values and practices into the health system to promote the provision of holistic care that is culturally acceptable to PLWE (WHO 2019).

The findings of this study show that healthcare professionals are aware of the cultural beliefs surrounding epilepsy in selected rural communities. For instance, some cultures perceive epilepsy as a spiritual or mystical phenomenon rather than a medical illness. Understanding these beliefs allows health professionals to provide management that supports the patient’s cultural values and preferences, which may significantly influence their experience and choice of treatment. Transcultural nursing acknowledges the significance of cultural competence in health care provision. Furthermore, it emphasises the necessity for health care providers to be sensitive to cultural beliefs, values and practices of PLWE. Health care providers should be able to identify cultural factors that influence how PLWE perceive epilepsy and their health-seeking behaviour. In this framework, researchers acknowledged the significance of integrating the transcultural nursing principles to provide culturally congruent care that is acceptable and relevant to PLWE. This involved the acknowledgement and understanding of diverse cultural beliefs, values and practices when providing care to PLWE.

Many healthcare professionals are open to collaborating with traditional healers and faith-based healers, provided they receive education on the scientific management of epilepsy. Similarly, traditional healers are willing to learn about Western approaches to epilepsy management, even suggesting the establishment of a referral system to facilitate the early diagnosis and management of epilepsy. These findings align with those of Keikelame and Swartz (2015), who emphasised that capacity building and the development of referral systems can be the key aspects of effective collaboration in epilepsy management. According to WHO (2013), traditional healers offer a culturally acceptable and less stigmatised approach to epilepsy management, consistent with the perceptions of PLWE regarding its causes.

**FIGURE 1:**
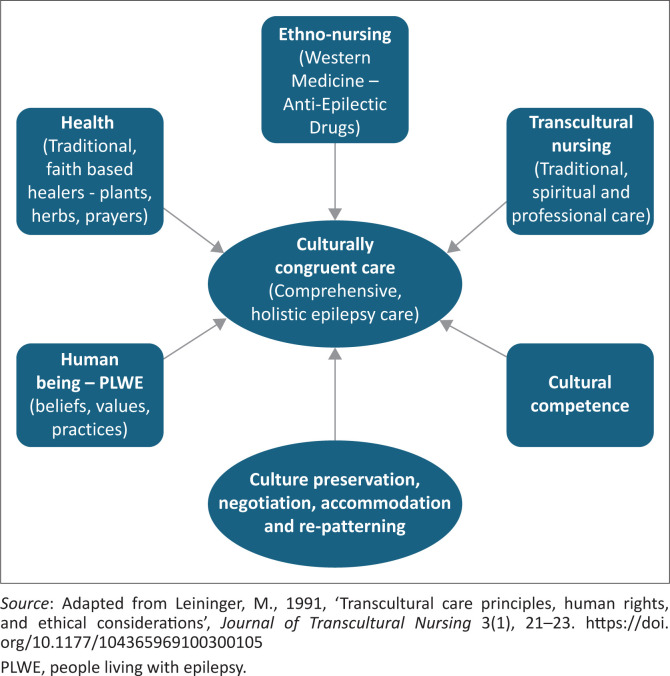
Conceptual framework: Cultural, faith-based and western medicine interventions.

This study found that health professionals are willing to engage in open communication with PLWE to gain insight into their diverse cultural backgrounds. Furthermore, awareness of cultural beliefs and values facilitates the establishment of culturally congruent management strategies that resonate with their beliefs and values. Notably, both traditional healers and faith-based healers expressed a keen interest in learning about developed medicine and collaborating with healthcare professionals. Health professionals need to be sensitive to these beliefs and provide suitable support and education while respecting the PLWE’s cultural perceptions (Zhu et al. 2023). Through understanding and integrating cultural beliefs and practices into their care plans, patient outcomes can be enhanced, thus promoting improved overall well-being among this population (Zhu et al. 2023). However, challenges faced by faith-based healers pose a significant concern, as they often struggle to understand traditional healers’ approach to managing epilepsy. Traditional healers are sometimes perceived as performing rituals that go against the beliefs of faith-based healers. This challenge was noted as a potential barrier that could hinder collaboration between the two groups. Nevertheless, open discussions can certainly help them understand and shift their perspective, potentially leading to mutual agreement. This study suggests that the conceptual framework (as depicted in Figure 1) is most likely to be effective in enhancing the quality of life of PLWE. As outlined in this discussion, these three groups require education on epilepsy and PLWE to promote mutual respect and understanding.

### Interventions to provide culturally congruent care

To ensure culturally congruent care for epilepsy, the implementation of culturally sensitive education programmes is required. These programmes should be tailored to meet the specific needs and beliefs of different cultural groups. This would include the establishment of interdisciplinary teams comprising health professionals, traditional healers and faith-based healers. These teams would work together to develop comprehensive care plans that incorporate both medical treatments and traditional healing practices. By combining their expertise, these practitioners can address the physical, emotional and spiritual needs of patients with epilepsy.

Culturally congruent care requires culture preservation, culture negotiation, culture accommodation and culture re-patterning as outlined next:

#### Culture preservation

Leininger (1991) emphasises the value of preserving customs and traditions that are fundamental to people’s identities and well-being. In order to support holistic healing, this entails appreciating the importance of these practices and implementing them into healthcare practices.

#### Culture negotiation

Culture negotiation refers to the process of identifying common ground between the patient’s beliefs, values and practices and those of the healthcare providers to ensure culturally congruent care. This process involves transparent communication, mutual respect and readiness to modify treatment plans to meet the patient’s cultural needs.

#### Culture accommodation

Culture accommodation involves modifying healthcare practices to meet the needs of PLWE from diverse cultural backgrounds. This may include modifying communication methods, treatment plans or care environments to ensure provision of culturally sensitive and acceptable care.

#### Culture re-patterning

Culture re-patterning is the process of assisting people in changing their cultural practices or beliefs to support improved health. This could entail teaching patients about different treatment options or motivating them to take up new behaviours that are consistent with evidence-based procedures. Leininger (1991) emphasises that people can improve their quality of life and general well-being by re-patterning culture.

Furthermore, by ensuring clear communication through bilingual staff members, PLWE would have a better understanding of their condition and how to manage it effectively. Engaging caregivers or community leaders in negotiations about treatment options and management strategies would help ensure that decisions are made in line with cultural values and preferences. Promoting patient-centred care through open communication and shared decision-making would also ensure culturally congruent care. Healthcare providers would actively listen to PLWE’s concerns, beliefs and treatment preferences, while providing accurate information about epilepsy management options. This approach would permit PLWE to actively contribute in their management choices, while respecting their cultural beliefs and values.

Adopting this approach, healthcare professionals would undergo comprehensive training in cultural competence, including understanding diverse cultural perspectives on epilepsy, beliefs regarding its causes and traditional healing practices. Continuous community engagement plays a crucial role in ensuring culturally congruent care for epilepsy. Collaborating with community leaders, religious institutions and local organisations would be instrumental in raising awareness about epilepsy within different cultures. Involving these stakeholders in educational campaigns and support groups would enable PLWE to access culturally appropriate resources tailored to their specific needs.

Training programmes should be developed to educate health professionals about various cultural practices related to epilepsy treatment. Similarly, traditional healers and faith healers should also receive training in modern medical approaches to enhance their understanding of epilepsy management. Involving faith healers and traditional healers in the care process would allow for a holistic approach to management that addresses both the physical and spiritual aspects of epilepsy. Health professionals should respect these beliefs while also educating PLWE about the importance of adhering to prescribed treatments.

Workshops would be necessary to capacitate the groups, enabling them to establish a strong working relationship and understand each other’s roles. This collaboration is poised to enhance current epilepsy management practices. Further research would determine contextual effectiveness and inform necessary interventions for improvement if warranted. Traditional healers should be encouraged to work closely with Western-trained practitioners to ensure compatibility between their treatments and developed medical interventions. Faith-based healers should offer emotional support while respecting the medical decisions made by Western-trained practitioners.

### Implications

The conceptual framework may assist care providers to have a better understanding of diverse cultural beliefs, values and practices hence enabling them to provide culturally congruent care as required by PLWE. Furthermore, it promotes provision of patient-centred care that respects the cultural diversity of PLWE. Healthcare practitioners can enhance patient trust and enhance health outcomes by recognising and integrating cultural beliefs and traditions into treatment regimens. Additionally, by fostering acceptance and understanding, this strategy lessens the stigma associated with epilepsy in rural communities.

Furthermore, a culturally congruent care framework can increase access to quality healthcare services for individuals living with epilepsy in rural areas. By understanding the cultural barriers that may prevent individuals from seeking or receiving care, healthcare providers can develop strategies to overcome these obstacles and ensure that all patients have equal access to necessary treatments. This ultimately leads to better health outcomes for individuals living with epilepsy in rural communities.

Moreover, a framework for culturally sensitive care can improve access to high-quality medical care. Healthcare practitioners can devise techniques to surmount cultural barriers that may impede individuals from seeking or getting care, thus guaranteeing equitable access to necessary treatments for all patients. In Ultimately, this improves the health of those who live with epilepsy in rural communities.

## Conclusion

Incorporating cultural beliefs, values and practices into medically developed approaches to the management of epilepsy is crucial for providing holistic, culturally congruent care for PLWE, while promoting diversity and inclusivity within society. By integrating these beliefs into healthcare delivery, health providers can offer care that is sensitive to PLWE’s preferences while still adhering to evidence-based management.

The conceptual framework for providing culturally congruent care highlights the importance of communication, respect and shared decision-making among all stakeholders involved in epilepsy management. The collaborative efforts of traditional healers, faith-based practitioners and Western-trained practitioners are essential in managing epilepsy effectively. This conceptual framework serves as a guideline for promoting effective communication and facilitating shared decision-making. Furthermore, the unnecessary delay in early diagnosis and treatment can be prevented as interventions will be tailored to accommodate cultural beliefs, values and practices hence improving the quality of life of PLWE.
